# Mining the past for the future: cyanobacterial herbarium specimens for systematics, evolution, and cyanotoxin diversity

**DOI:** 10.3389/fmicb.2026.1827668

**Published:** 2026-05-14

**Authors:** Petr Dvořák, Dale A. Casamatta

**Affiliations:** 1 Department of Botany, Faculty of Science, Palacký University Olomouc, Olomouc, Czech Republic; 2 Department of Biology, University of North Florida, Jacksonville, FL, United States

**Keywords:** cyanobacteria, cyanotoxins, genomics, herbarium collections, systematics

## Abstract

While cyanobacteria are some of the most important primary producers on Earth, we still have limited understanding of their diversity and evolution. Herbarium collections offer a large and almost untapped source of cyanobacterial specimens. Here, we summarize the application of omics in the study of herbarium specimens of cyanobacteria which can enable us to advance our understanding of the diversity and evolution of this lineage. While we focus on taxonomic studies, where herbaria specimens have already uncovered novel diversity and resolved long-standing taxonomic conundrums by genome analysis of the type specimens, other uses abound. We show that herbaria can serve as instruments to investigate the impact of global changes based on amplicon sequencing. Further, researchers are discovering that cyanotoxins remain stable in the dried specimens, allowing for chemical analyses and novel compound discovery and exploration of past patterns in the cyanotoxin diversity in the environment. Ultimately, we would like to motivate researchers to utilize herbaria collections by outlining some of the possibilities of cyanobacterial-based herbaria samples.

## 1 Introduction

Herbarium collections as dried plants mounted on paper sheets date to 16th century Bologna and Ferrara (modern day Italy). At that time, plants were primarily collected and stored for medicinal purposes. It was Luca Ghini, considered the father of the modern herbarium collections, who developed the basic methods for plant preservation and gathered large herbarium collections (Cristofolini, 2024). Today, there are 4,749 herbarium (or *hortus siccus*) collections in the world (accessed on June 3rd, 2025; Index Herbariorum, https://sweetgum.nybg.org/science/ih/). Containing millions of specimens of plants, fungi, algae, cyanobacteria, and other organisms, the Global Biodiversity Information Facility (www.gbif.org) contains records of 146,679,188 preserved specimens (as of July 8th, 2025).Note that preserved specimens are not always dried samples and www.gbif.org do not contain all herbarium collections. Regardless of actual specimen number, herbarium collections represent a vast trove of historical data. Traditionally, papers published utilizing herbarium collections chiefly focused on taxonomy, history, herbarium resources, herbarium protocols, biodiversity, and biogeography. As expected, most works center on vascular plants (59.8%), with other organisms less frequently investigated (e.g., ascomycetes (3.9%), bryophytes (3.4%), ochrophytes (0.9%), and cyanobacteria (0.1%), *sensu* (Marín-Rodulfo et al. 2024). However, herbaria samples can also be used to address current issues in biology. For example, (Ng et al. 2023) employed herbaria samples to examine changes in leaf traits of poison ivy (*Toxicodendron radicans*) over the last 150 years. (DeLeo et al. 2019) used herbaria samples to document changes in the physiology and phenology of *Arabidopsis thaliana* as a result of climate change. Thus, these “preserved” samples can continue to provide useful information to science.

**Figure 1:**
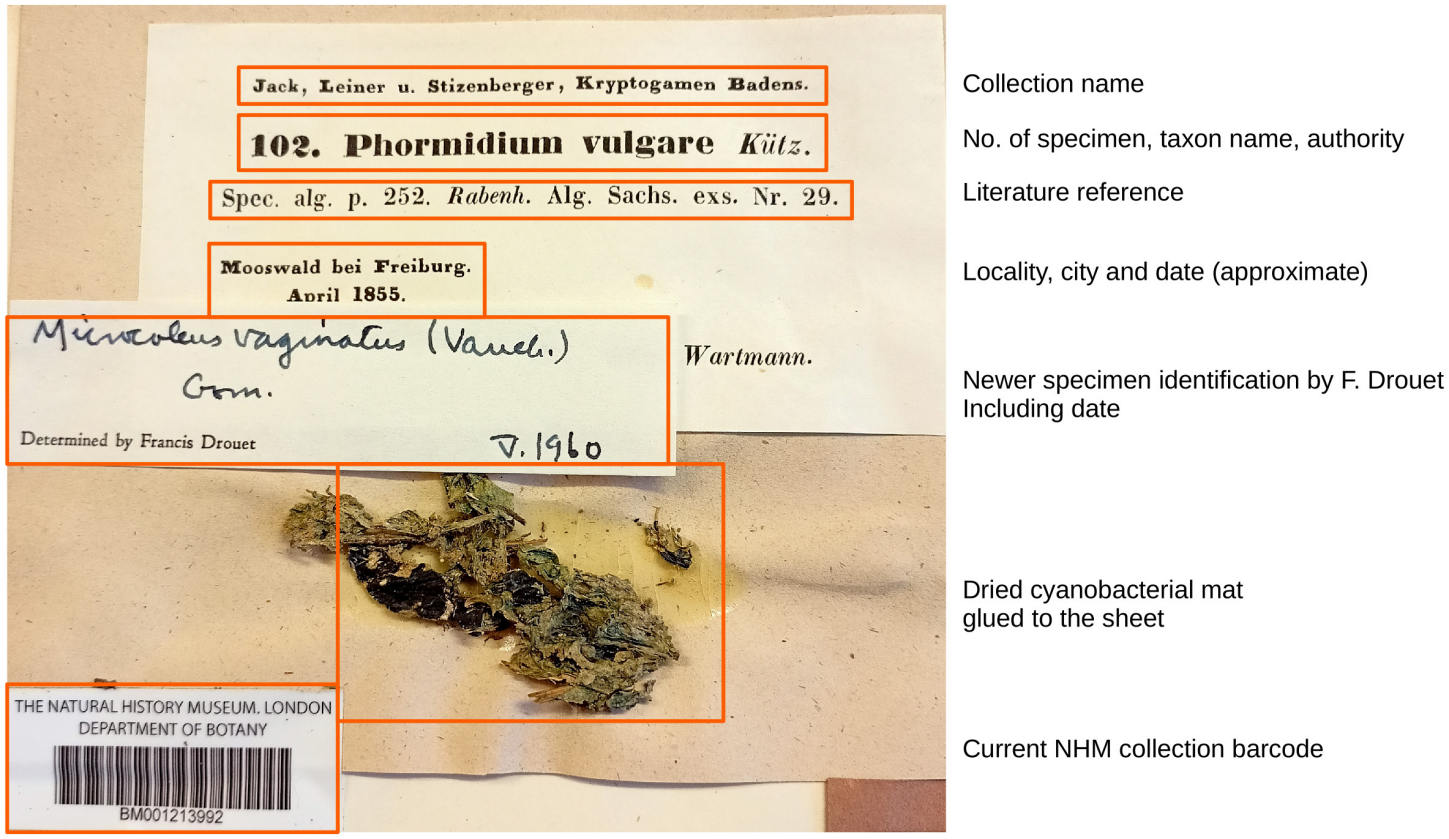
An example of herbarium specimen deposited in NHM (London, UK). Aside from dried, glued mat material on a paper sheet, cyanobacterial biomass can also be stored in paper envelopes glued to sheets, filtered on paper (often for planktic specimens), or have the biomass just stick to the paper due to the polysaccharides in sheaths. The label compositions differ over time periods and conventions in the particular collections. However, labels typically contain most of the information depicted in the figure. Some specimens in the Botanic Garden and Botanical Museum (Berlin, Germany) contain hand-written morphological descriptions on the label.

Cyanobacteria are among the most important primary producers on the Earth and thrive in myriad habitats if there is sufficient light (Whitton, 2012). Due to their morphological and ecological similarity to algae, the nomenclature for cyanobacteria has been governed by the International Code of Nomenclature for algae, fungi, and plants (ICN; https://www.iapt-taxon.org/nomen/main.php). As such, cyanobacteriologists have gathered samples and deposited specimens into collections similar to botanists; a total of 198,465 preserved specimens from around the globe during the last 200 years (www.gbif.org; 8th July 2025). Note that this is likely an underestimate since some herbarium collections may not have cataloged all the specimens stored in their collection. In any case, cyanobacterial herbarium collections contain a large and almost untapped source of data to study questions of taxonomy, evolution, biogeography, and biodiversity of cyanobacteria in a historical context (see an example of the cyanobacteria herbarium specimen in Figure 1).

Cyanobacterial taxonomy and identification was established by using morphological features prior to the advent of molecular methods. Subsequent phylogenetic analyses based on the 16S rRNA gene and whole genomes have revealed that cyanobacterial evolution is laden with instances of convergent evolution; the same morphotypes have separately evolved many times (*e.g*., taxa looking like the “simple” bacilloid genus *Synechococcus* likely evolved at least 12 times, *sensu* Dvořák et al., 2014). Morphological features are often not sufficient to recognize and identify species, and the presence of cryptic species has long been acknowledged (Casamatta et al., 2003). Likewise, phenotypic plasticity can make accurate identification challenging, with some features only being observed under particular conditions (e.g., “nodule” forming filaments *sensu* Perkerson et al., 2011). Many cryptic species can be recognized using single marker sequencing (*e.g*., the 16S−23S rRNA gene), but increasingly frequently by the whole genome sequencing [reviewed in (Dvořák et al., 2015, 2023, 2024; Skoupý et al., 2024)]. However, many cyanobacterial specimens may not be correctly identified since the vast majority of them were identified based solely on morphology.

(Marín-Rodulfo et al. 2024) found only three publications employing herbarium collections of cyanobacteria from 1842 to 2022. However, this figure is likely an underestimate since it does not account for newly described taxa throughout this period. The words “herbarium” or “herbaria” are not usually mentioned in the title and keywords, only in the species descriptions, which cannot be searched by Scopus used by (Marín-Rodulfo et al. 2024). The vast majority of the *ca*. 200,000 cyanobacterial specimens were likely stored and never investigated again. In the past, taxonomists may have listed type materials of taxon of particular interest, but they may not have investigated the specimen rigorously (e.g., Gomont, 1892). Only occasionally did taxonomists visit herbarium collections and investigate and re-identify specimens based on the current taxonomic discourse at that time. For example, Francis Drouet (1907–1982) visited herbarium collections in Germany (Botanic Garden and Botanical Museum, Berlin), the United Kingdom (Natural History Museum, London), and other places, re-evaluating the specimens and noting new specimen identifications directly on the labels, which unfortunately did not necessarily get published (P. Dvořák, personal observation in herbarium collections).

In this paper, we will review past and current work performed on cyanobacterial herbarium specimens involving omics techniques. We will explore future possibilities of these specimens in taxonomy, diversity, evolution, and ecology studies utilizing omics. Ultimately, we would like to motivate the community of cyanobacteriologists to more fully engage with herbarium specimens in their studies and add to the growing store of specimens.

## 2 Diversity and taxonomy based on gene and genome sequences from herbaria

Although Drouet made cyanobacterial herbarium mining infamous, actual genetic analysis was pioneered by (Palińska et al. 2006). They sequenced incomplete 16S rRNA genes of seven specimens, performed phylogenic analyses, and characterized morphologies using light and electron microscopy. Specimens were selected from some common, cosmopolitan genera including *Microcystis, Chroococcus, Microcoleus* (technically, it was species *M. chthonoplastes*, later renamed as *Coleofasciculus sensu* (Siegesmund et al., 2008), *Trichodesmium*, and *Nostoc*. Specimens were acquired from the herbarium collection of the Institute of Botany, University of Vienna (Austria), the Swedish Museum of Natural History (Stockholm, Sweden), and a personal collection of S. Golubić and represented samples collected between 1850 and 1990. The recovered 16S rRNA sequences had between 783 and 1,210 bp, suggesting relatively low fragmentation of the DNA. It should be noted that only a single PCR product was sequenced per herbarium specimen (without cloning). However, cyanobacterial specimens are often a consortium of many species and some taxa, such as *Nostoc*, are known to have multiple ribosomal operons (Carmona Jiménez et al., 2023). This lack of sequencing diversity is likely the result of the sequencing technologies at the time. While this paper was clearly breakthrough in the field of hDNA (historical DNA) analysis, it did not motivate cyanobacteriologists to further intensive work with the herbarium specimens.

The first genome sequence of herbarium preserved cyanobacterium was obtained by (Dvořák et al. 2020). Two specimens of *Nostoc* (labeled as *N. commune* and *N. flagelliforme*; dated to 1914 and 1974 respectively) were sequenced using Illumina technology. *Nostoc* is one of the most diverse and most widely distributed cyanobacteria (Fidor et al., 2019). It is characterized by large and prominent colonies growing on soil. Due to its conspicuous nature and ease of collection, large amounts of biomass are often deposited in herbarium collections, allowing enough material for DNA sequencing. Specimens were obtained from the Botanic Garden and Botanical Museum (Berlin, Germany). Each herbarium specimen proved a consortium of microbial species (a metagenome) consisting of one metagenome assembled genome (MAG) of *Nostoc*, one accompanying cyanobacterial MAG of cf. *Scytonema*, and several alphaproteobacterial MAGs. The *Nostoc* genome assemblies were fragmented (1,554 and 2,360 contigs) and phylogenomic inference confirmed the samples were *Nostoc*. This work unlocked the potential of the herbarium as a source of material for the discovery of the unknown cyanobacterial genomic diversity. A year later, (Jungblut et al. 2021) published one high-quality draft genome of *Nostoc* obtained from the Natural History Museum (NHM; London, UK). The material was collected during the British Arctic Expedition (1875–1876), expanding the range of possible dates for sequencing of herbarium specimens. They also sequenced a recent herbarium specimen (from 2015) for comparison, and again employed Illumina technology. The genome analyses revealed large accessory genome and extensive secondary metabolite production potential. Both mentioned papers represent a proof-of-concept for herbarium sequencing.

Collections of the NHM (London, UK) played a crucial role in the largest genome sequencing effort of cyanobacterial herbarium specimens to date. NHM has a large collection of specimens of the nearly ubiquitous, cosmopolitan genus *Microcoleus*. As with *Nostoc, Microcoleus* inhabits soils in temperate regions, but also thrives in inhospitable habitats including hot and cold deserts. *Microcoleus* is a common terrestrial pioneer taxon, forming soil crusts and facilitating the habitat for other organisms (Belnap and Lange, 2001; Nelson et al., 2022). (Stanojković et al. 2024) sequenced eight genomes of *Microcoleus* deposited at NHM and added them to additional sequences, genomes, and MAGs from GenBank (https://www.ncbi.nlm.nih.gov/). Phylogenomics and population genomic analyses revealed that *Microcoleus* has diverged into the speciation continuum composed of a range of species with a variable gene flow between them. In a related study, phylogenomic and morphological analyses revealed that the four herbarium specimens represented undescribed species of *Microcoleus* and the rest was classified within one of the investigated putative species (Skoupý et al., 2024). This suggests that herbarium specimens, the oldest of which was possibly 200 years old, can also be a source to uncover unknown diversity or perhaps extinct lineages, because no related extant sequenced genomes matched the historic depositions. Moreover, one of the specimens was deposited by Desmaziéres as a type for *Microcoleus*, and later confirmed by (Gomont 1892). Thus, for the first time, a taxonomic revision based on herbarium-derived genomes and type specimens was performed (Skoupý et al., 2026).

The first amplicon sequencing of herbarium specimens was performed in NHM as well. Jungblut & Hawes (2017) conducted a comparison of cyanobacteria in historic microbial mats from Ross Island and McMurdo Ice Shelf, collected during Captain R. F. Scott's Discovery Expedition (1902–1903), with modern samples from the same areas. Studies have indicated that the most pronounced impacts of climate change are likely to impact polar regions (Collins et al., 2024). However, few long-term observations of microbial communities exist to actually assess natural variation. Using 16S rRNA gene surveys, Jungblut & Hawes found that historic and modern cyanobacteria assemblages were dominated by the same genotypes, with some variation (Jungblut and Hawes, 2017). Modern communities showed higher richness with additional genotypes but maintained high similarity to other Antarctic cyanobacteria. These results indicate slow genotype turnover and considerable community stability in Antarctic microbial mats, suggesting resilience to future climate-driven environmental change.

## 3 Cyanotoxins in herbarium specimens

Cyanobacterial abundance and persistence has been shown to be positively correlated with anthropogenic changes to the environment (Paerl and Huisman, 2009; Doubek et al., 2015). Cyanobacteria produce a myriad assortment of secondary metabolites, most significantly cyanotoxins (Codd, 1995; Li et al., 2023). However, it has not been determined if such cyanotoxins remain stable in the dried specimens for a long time. (Metcalf et al. 2012) analyzed 30 cyanobacterial herbaria specimens (1839–1950) from the NHM again; this time for the presence of microcystins. The specimens were sampled primarily in the UK, but also from Europe, the Americas, and Africa. Microcystins were detected in 46% of samples by high performance liquid chromatography, 83% of samples by immunoassay, and 97% of samples by mass spectrometry. However, the *mcy*D microcystin marker was confirmed in only in 17% of samples using PCR.

A second paper extended this approach to seven cyanobacterial mats collected in Antarctica during Captain Scott's 1901–1904 expedition (Jungblut et al., 2018). Microcystins were detected in six samples, β-N-methyl-amino-L-alanine was detected in one sample, and no sample contained anatoxin-a. These findings demonstrate that microcystins and other cyanotoxins can persist for *ca*. 170 years in dried material, validating historic herbarium collections as archives for retrospective ecotoxicological and evolutionary studies of cyanotoxin production irrespective to the environment of origin. However, it must also be noted that there exists the possibility that the detected microcystins may have originated from another source from which the materials were collected. More systematic studies are needed to further develop this topic beyond simple presence/absence of the cyanotoxins. How long cyanotoxins in general (other than microcystins) remain measurable, though, remains to be assessed. (Chollet-Krugler et al. 2019) note that mycosporine-like amino acids (MAAs) may degrade in herbaria samples based on how the samples were archived, so it remains possible that the toxins are persistent but just not stored in a manner conducive to detection in the long-term. Chemical compounds remained stable in the plant herbarium specimens as well (e.g., Foutami et al., 2018), providing evidence that mining herbaria samples for novel chemically bioactive compounds has great potential.

## 4 Limitations of historical herbarium research in cyanobacteria

We have shown that cyanobacterial herbaria samples may provide an untapped resource for biological and chemical data, but there are some caveats. One note of caution concerns how herbarium specimens are dried before deposition. Some are dried slowly at lower temperatures, others fast with higher temperatures, some augmented with silica gel, alcohol dried, microwaved, etc. These approaches differentially fragment DNA and therefore affect the sequencing effectivity (e.g., Forrest et al., 2019). Purines may be lost due to heat causing deamination, leading to spurious C → T mutations (reviewed and tested in plant herbariums by (Staats et al. 2011)). Moreover, the efficacy of DNA extraction and sequencing may be lowered by chemical treatments such as HgCl_2_, which was often employed in the past, but whose effect has not yet been rigorously tested. Most herbaria specimens are not kept in sterile conditions, which may attract contaminant organisms such as bacteria, fungi, protists, and animals, that can either destroy the specimen or contaminate the DNA for the sequencing. Herbarium sequencing may also be logistically difficult due to the relatively low amount of biomass in specimens, and thus DNA extraction yields can be minimal. In some collections, the treatment processes are unknown as records may have been lost during floods, fires (e.g., fire after bombing in Berlin during WWII), other catastrophic events, and moving.

Most of these limitations are shared between cyanobacteria and plants, but there are some specific concerns which deserve our attention. For example, the unclear taxonomic status of some specimens and immense potential cryptic diversity of some cyanobacterial species (cryptic diversity is reviewed in Dvořák et al., 2015, 2023, 2024) may both present significant impediments. Indeed, plant herbaria contain erroneously identified specimens, but this problem may be more extensive in cyanobacteria. An excellent example of this concerns the previously addressed genus *Microcoleus*. It is difficult to estimate how many specimens of *Microcoleus* are deposited in collections since the morphological circumscription of “*Microcoleus*” has undergone extensive revision. The overall cell morphology overlaps with the taxonomically rich “*Phormidium*” groups VII and VIII (classification *sensu* Komárek and Anagnostidis, 2005), and they share a common ancestor (Hašler et al., 2012). Thus, herbarium specimens have been deposited as various species from *Microcoleus* and *Phormidium* (see (Skoupý et al. 2024) for details). At one point, sheath characteristics were considered very significant in identifications for this lineage, but phylogenetic reconstructions using 16S data show that they are not particularly robust characters due to plasticity and culturable inducement. Further, sheaths do not preserve well, so even if the original samples had them, they may be lost to subsequent investigators.

Cyanobacterial herbarium specimens are actually a consortium of microbes (e.g., both the photosynthetic cyanobacteria but also potentially extensive co-existing prokaryotes), and therefore metagenomes. Cyanobacteria have long been known to select for different heterotrophic bacterial communities (Casamatta and Wickstrom, 2000), leading to additional sequencing issues. Each specimen may also contain different species of cyanobacteria, and potentially different genotypes of each species, which complicates sequencing and subsequent downstream analyses. Furthermore, it may be difficult to find the species of interest stated on the label in the herbarium specimen. The phycologist depositing the specimen might have put on a label a species which could have been rare in the sample. If there were morphologically similar or cryptic species, how can we decide which DNA sequence belongs to what material? By way of example, the previously noted specimens of *Microcoleus* have usually only one cyanobacterial genotype and almost no other bacterial species (Stanojković et al., 2024). Thus, this problem may not be so significant as it seems, or perhaps it is dependent on the environment of sampling and taxon of interest. In any case, this issue needs to be kept in mind for the future herbarium users. Finally, the cyanobacterial herbaria depositions are usually much smaller than plants, although some quite large specimens of *Nostoc* exist (Jungblut et al., 2021). Thus, as small amount as possible should be destroyed, which can also hinder the yield of the DNA extraction. Small amounts of the DNA can be extracted using ancient DNA extraction protocols (see application of protocols by (Kistler 2012) and (Meyer and Kircher 2010) in (Stanojković et al. 2024)).

## 5 The future—opportunities of the herbarium research in cyanobacteria

Studies of the herbarium specimens of cyanobacteria are still rare, but we would like to inspire cyanobacteriologists to use the collections and ask research questions involving herbarium specimens. Of course, cyanobacterial collections are less extensive and typically smaller compared to plants, and therefore a narrower portfolio of potential research exists. Likewise, morphological investigations, which tend to remain stable in plants, may not be as robust with cyanobacteria. That noted, herbaria resources may help shed light on such disparate topics as:

- Novel species—the species diversity of cyanobacteria is extensive (Guiry and Guiry, 2025), but based on the number of species and other taxa published every month, we can assume that the diversity is much higher. Herbarium collections represent a great source to discover species from disparate habitats and geographical areas. Many herbarium specimens were stored during expeditions in 19th and 20th century (for example see Captain Scott expedition above), potentially yielding novel taxa.
- Taxonomy—the analyses of type specimens to resolve taxonomic status of the current taxa by investigating the historical types. Using genome sequencing, only one new putative species has been documented in (Skoupý et al. 2024), but many questions remain unanswered. For example, some taxonomically confusing/challenging lineages (e.g., *Nostoc*) remain unresolved. These methods have been employed to determine the species identity of the now extinct angiosperm *Armeria arcuata* (Abeli et al., 2025), and may prove a boon to cyanobacterial researchers.
- Novel chemical compounds—cyanotoxins may remain stable over long time periods (see above). Historical patterns of the cyanotoxin presence in investigated localities can be investigated. For example, the recently described *Aetokthonos hydrillicola* has been implicated in bald eagle mass mortalities by causing vacuolar myelinopathy (Breinlinger et al., 2021); has this toxin been present before or is it a recent advent?
- Environmental changes. Some algae, such as coccolithophores or diatoms, have long been employed for paleo-reconstructions (e.g., (Stoermer et al. 1996)). Why not cyanobacteria? If extensive enough samples exist, questions pertaining to shifts in species concurrent with climate change or anthropogenic alterations may be answered. While paleo-reconstructions such as seen with frustules may not be reasonable due to lack of diagnosable, stable structures (e.g., the silicaceous remains of diatoms), features like the number and placement of heterocytes, general taxa present, and potentially even cyanotoxins present may be reasonably employed. For example, the presence of akinetes can be used to estimate past trophic conditions and to estimate nitrogen budgets (Findlay et al., 1994, 1998). As an added bonus, a whole community of microbes can be investigated, not just cyanobacteria.
- Genome evolution—questions relating to mutations, structural variation across the genomes in time, across populations, and across species can all be addressed. Historical and current populations from the same locality can be compared. How fast do cyanobacteria evolve? Is a century period long enough to observe speciation?
- Funding support—herbaria are expensive to establish and maintain, and many rely on the employment of their materials for future funding (similar to botanical collections or gardens). Thus, investigations using such materials may have a pragmatic aspect: their employment in answering scientific questions helps justify these valuable resources.
